# Skeletal muscle adaptations to high‐intensity, low‐volume concurrent resistance and interval training in recreationally active men and women

**DOI:** 10.14814/phy2.15953

**Published:** 2024-03-15

**Authors:** Adam J. Sterczala, Nathaniel Rodriguez‐Ortiz, Evan D. Feigel, Kellen T. Krajewski, Brian J. Martin, Nicole M. Sekel, Mita Lovalekar, Christopher K. Kargl, Kristen J. Koltun, Carola Van Eck, Shawn D. Flanagan, Christopher Connaboy, Sophie L. Wardle, Thomas J. O'Leary, Julie P. Greeves, Bradley C. Nindl

**Affiliations:** ^1^ Neuromuscular Research Laboratory and Warrior Human Performance Research Center University of Pittsburgh Pittsburgh Pennsylvania USA; ^2^ Department of Orthopaedic Surgery University of Pittsburgh Pittsburgh Pennsylvania USA; ^3^ Army Health and Performance Research Army Headquarters Andover UK; ^4^ Present address: Human Engineering Research Laboratories VA Pittsburgh Healthcare System Pittsburgh Pennsylvania USA; ^5^ Present address: Center for Lower Extremity Ambulatory Research Rosalind Franklin University of Medicine & Science North Chicago IL USA; ^6^ Present address: Norwich Medical School, Faculty of Medicine and Health Sciences University of East Anglia Norwich UK; ^7^ Present address: Division of Surgery and Interventional Science University College London London UK

**Keywords:** capillarization, concurrent training, fiber type, hypertrophy, resistance training

## Abstract

This study compared the structural and cellular skeletal muscle factors underpinning adaptations in maximal strength, power, aerobic capacity, and lean body mass to a 12‐week concurrent resistance and interval training program in men and women. Recreationally active women and men completed three training sessions per week consisting of high‐intensity, low‐volume resistance training followed by interval training performed using a variety upper and lower body exercises representative of military occupational tasks. Pre‐ and post‐training vastus lateralis muscle biopsies were analyzed for changes in muscle fiber type, cross‐sectional area, capillarization, and mitochondrial biogenesis marker content. Changes in maximal strength, aerobic capacity, and lean body mass (LBM) were also assessed. Training elicited hypertrophy of type I (12.9%; *p* = 0.016) and type IIa (12.7%; *p* = 0.007) muscle fibers in men only. In both sexes, training decreased type IIx fiber expression (1.9%; *p* = 0.046) and increased total PGC‐1α (29.7%, *p* < 0.001) and citrate synthase (11.0%; *p* < 0.014) content, but had no effect on COX IV content or muscle capillarization. In both sexes, training increased maximal strength and LBM but not aerobic capacity. The concurrent training program was effective at increasing strength and LBM but not at improving aerobic capacity or skeletal muscle adaptations underpinning aerobic performance.

## 1 INTRODUCTION

Military training must prepare servicemen and women for a diverse range of physical tasks, the most arduous of which are load carriage, casualty evacuation, and manual materials handling (maximal and/or repeated lifting and carrying) (Vaara et al., 2022). Performance of these tasks relies on sufficient strength, power, lean body mass, endurance, and aerobic capacity (Angeltveit et al., 2016; Cocks et al., 2016; Fallowfield et al., 2012; Hauschild et al., 2017; Hydren et al., 2017). Thus, to prepare personnel for military occupational demands, physical training must stimulate muscle hypertrophy while increasing performance across the spectrum of physical fitness domains.

Concurrent training—simultaneous resistance and aerobic training—elicits muscle fiber hypertrophy (Lundberg et al., 2022), muscle fiber type shifts (Tsitkanou et al., 2017), increases markers of mitochondrial biogenesis (MacNeil et al., 2014) and increases muscle capillarization. These physiological adaptations contribute to improved strength, power, muscle endurance, and aerobic performance, and therefore, may be an ideal training stimulus to improve military occupational performance in men and women (Kraemer et al., 2004; Nindl et al., 2017). As concurrent training includes both resistance and aerobic training, exercise volume is likely to be greater than single‐mode training. Given that high training volume may increase the risk of musculoskeletal injuries in military populations employed in physically arduous roles (Almeida et al., 1999; Jones & Knapik, 1999), concurrent training for military personnel should seek to limit training volume. Recent meta‐analyses have demonstrated that resistance training intensity is the key determinant of strength adaptations (Carvalho et al., 2022; Currier et al., 2023; Grgic, 2020), whereas muscle hypertrophy is highly dependent on resistance training volume (Currier et al., 2023). As a result, high‐intensity, lower‐volume resistance training may be ideal for eliciting strength and power gains with modest, although potentially sub‐optimal, muscle hypertrophy. Additionally, lower volume interval training can elicit similar aerobic performance improvements compared to higher volume, continuous moderate intensity aerobic training (Foster et al., 2015; Haddock et al., 2016; Macpherson et al., 2011). Thus, low‐volume, high‐intensity concurrent training may provide a means to prepare military personnel for occupational demands while mitigating the risk of overuse‐associated injuries.

Previously, we demonstrated that low‐volume, high‐intensity concurrent training improves military occupational and physical performance in recreationally active, enlistee aged men and women (Sterczala et al., 2023). As these military tasks simultaneously rely on multiple fitness domains, analysis of skeletal muscle pre‐ and post‐concurrent training could yield insights into the adaptations underpinning military performance improvements. Muscle adaptation analyses could also identify how the program could be improved to optimize fitness in military personnel. Additionally, past research has reported differences between sexes in muscle fiber cross‐sectional area (Staron et al., 2000), muscle fiber type (Staron et al., 2000), capillarization (Keteyian et al., 2003; Ross et al., 2023), and mitochondrial density (Arribat et al., 2019; Broskey et al., 2014) which may confer advantages in strength (Roberts et al., 2023) and endurance‐based (Schiaffino & Reggiani, 2011) tasks. As militaries are employing gender‐neutral fitness tests, ensuring that the training prepares both sexes for the demands of these tests and their military roles is essential. Analysis of muscle adaptations to the concurrent training program could determine whether the men and women responded similarly, or if sex‐specific training prescription modifications are needed to optimize hypertrophic, angiogenic, and aerobic adaptations.

The purpose of this study was to determine the effects of a 12‐week resistance training with a concurrent interval training exercise program, on skeletal muscle outcomes in men and women. Muscle fiber type, fiber cross‐sectional area, markers of mitochondrial biogenesis, and muscle capillarization were studied before and after the training intervention. We hypothesized that men and women would demonstrate similar muscle fiber hypertrophy. Additionally, we hypothesized that training would elicit a transition to a more fatigue resistant, oxidative muscle fiber type distribution, increase mitochondrial biogenesis biomarker content and increase capillarization consistent with previous research, and that these adaptations would not differ between sexes.

## 2 METHODS

### 2.1 Study design

Data presented are part of the UK Ministry of Defense (WGCC 5.5.6‐Task 0107) supported Soldier Performance and Readiness as Tactical Athletes (SPARTA) study. The SPARTA study sought to improve military relevant performance in men and women in the British Army's newly developed, gender‐neutral Role Fitness Test for Soldiers (RFT(S)). The purpose of the skeletal muscle analyses is to determine the physiological adaptations underpinning military and physical performance improvements. Before and after the 12‐week training intervention, participants completed physical performance testing and muscle biopsies across multiple testing visits. Aerobic capacity testing and body composition analysis was performed at visit 1, maximal strength testing at visit 2, RFT(S) performance at visit 3 and the muscle biopsies at visit 4. The minimal rest scheduled between testing visits was as follows: 24 h between visits 1 and 2, 48 h between visits 2 and 3 and 72 h between visits 3 and 4.

### 2.2 Participants

Participants included recreationally active individuals who reported exercising at least three times per week for at least 30 min per exercise session. Participants were free of any musculoskeletal injuries or conditions that could impair physical performance or musculoskeletal adaptations to exercise training. Prior to enrollment, all volunteers were informed of the risks and provided verbal and written informed consent. The investigation was approved by the University of Pittsburgh Institutional Review Board and United Kingdom Ministry of Defence Research Ethics Committee (903/MODREC/18).

### 2.3 Training intervention

**FIGURE 1:**
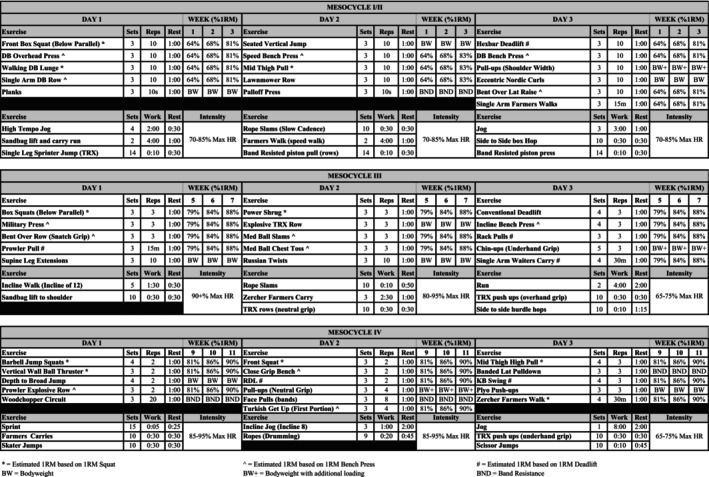
Concurrent resistance and interval training program.

All participants completed a 12‐week periodized concurrent resistance and interval training program consisting of 3 training sessions per week for a total of 36 training sessions. Training sessions were approximately 60–90 min in duration. Each session began with a dynamic warmup, followed by resistance training. After resistance training was complete, brief rest was provided after which interval training was performed. All training sessions were supervised by a National Strength and Conditioning Association Certified Strength and Conditioning Specialist. The resistance training program consisted of four mesocycles: (1) general physical preparedness (2 weeks), (2) preparation for peak force production (1 week), (3) peak force development (3 weeks) and (4) rate of force development (3 weeks). Set ranges, repetition ranges, and relative loading intensities for the four mesocycles are reported in Figure 1. A deload week followed the second, third, and fourth mesocycles to allow for adequate recovery and prevent overtraining. During deload weeks, training intensity was reduced by 50%. At the first and third visits of each deload week, participants were familiarized with the next mesocycle's exercises. During the second visit of each deload week, one‐repetition maximums (1RMs) for squat, bench, and deadlift were tested. Exercise selection focused on multi‐joint, complex movements including variations of squats, deadlifts, lunges, jumps, loaded carries, presses, pullups, and Olympic lift derivatives. Interval training exercise modalities contrasted from the literature primarily using cycling or running, instead employing variations of runs/sprints, jumps, upper and lower body bodyweight exercises, and loaded carries to more closely mimic military common and functional tasks. Interval training during the first and second mesocycles was performed at 70%–85% of age‐predicted maximal heart rate (HR_max_). For the third and fourth mesocycles, interval training intensity was performed at >80% HR_max_ for the first two sessions each week, while the third training session was performed at a lower intensity (65%–75% HR_max_). During interval training, HR was assessed with a chest mounted heart rate monitor (Garmin, Olathe, KS). Training staff monitored participants HRs throughout interval training and adjusted the rate at which the movements were performed to keep participants within the prescribed HR ranges. The full resistance and interval training programs are presented in Figure 1. Participants were instructed to abstain from excessive physical activity outside of training sessions and maintain their pre‐study dietary routine throughout the study. Participants were required to maintain a minimum of 95% training session compliance. Average training session compliance for the study was 98.8%.

### 2.4 Muscle biopsy

Vastus lateralis muscle biopsies were collected at rest, before and after the training intervention. Prior to the muscle biopsy visits, participants fasted overnight, for at least 8 h, and abstained from exercise for a minimum of 72 h. Biopsies were collected from the muscle belly under local anesthesia using a 6 mm Bergstrom biopsy needle with suction, as previously described (Tarnopolsky et al., 2011). Lidocaine administration remained superior to underlying fascia to avoid injection into muscle belly. Immediately following the biopsy, visible connective and adipose tissue was removed. A ~100 mg portion of muscle tissue was mounted cross sectionally on a cork board in a mixture of gum tragacanth and optimum cutting temperature mounting medium. The mounted tissue was snap‐frozen in liquid nitrogen cooled isopentane and immediately stored at −80°C until analysis. The remaining muscle tissue was portioned into ~30 mg aliquots and frozen in liquid nitrogen and stored in cryovials at −80°C until analysis.

### 2.5 Western blot

Tissues were lysed in T‐PER (Thermo Scientific, 78510) supplemented with HALT protease and phosphatase inhibitor cocktail (Thermo Scientific, 78444), and homogenized using a Bullet Blender Tissue Homogenizer (Next Advance, BBX24) at 4°C. After homogenization, lysates were centrifuged at 14,000*g* for 5 min at 4°C. Supernatant protein concentration was quantified using a bicinchoninic acid (BCA) protein assay (Thermo Scientific, cat# 23227) and then stored at −20°C. Loading samples were prepared using supernatants and Laemmli sample buffer (Bio‐Rad, 1610747) with 2‐mercaptoethanol then heated at 95°C for 5 min. In total, 25 μg of protein was loaded to a 4%–15% Mini‐PROTEAN TGX Stain‐Free Protein gel (Bio‐Rad, 4568083). Electrophoresis was performed at 70 V for 15 min, followed by 130 V for 50 min. Gels were transferred to LF‐PVDF membrane (Bio‐Rad, 1704275) for 7 min at 2.5A on a Trans‐Blot Turbo Transfer System (Bio‐Rad, 1704150). Membranes were then blocked for 5 min using EveryBlot Blocking Buffer (Bio‐Rad, 12010020) at room temperature with gentle rocking. Immunoblotting was performed overnight at 4°C using the following antibodies: PGC‐1α (1:1000; abcam 191838), Citrate Synthase (1:1000; CST 14309), and COX IV (1:1000; abcam, 33985). Membranes were washed five times for 5 min with tris‐buffered saline with 0.1% Tween 20 (TBS‐T). Membranes were the incubated for 1 h at room temperature with StarBright Blue 520 Goat Anti‐Mouse IgG (1:2500; Bio‐Rad, 12005867) and StarBright Blue 700 Goat Anti‐Rabbit IgG (1:2500; Bio‐Rad, 12004162). Following the incubation, membranes were washed in TBS‐T three times for 5 min each wash. Membranes were then air dried and imaged using a ChemiDoc MP (Bio‐Rad, 17001401). Images were quantified using Image Lab software from Bio‐Rad. Total PGC‐1α, citrate synthase, and COX IV content were normalized to total protein within the respective lane.

### 2.6 Immunohistochemistry

Frozen muscle tissue was sectioned (7 μm) using a cryostat (Leica, CM1860) and air‐dried for several hours prior to staining. To assess fiber type specific CSA and fiber type distribution, sections were rehydrated by washing three times with phosphate‐buffered saline (PBS) for 5 min and incubated for 90 min with an antibody solution containing the following antibodies: BA.D5 IgG2b (1:100; Developmental Studies Hybridoma Bank (DHSB), BA.D5‐c) for Type 1 muscle fibers, SC.71 IgG1 for Type IIa muscle fibers (1:50; DHSB, SC.71‐supernatant), 6‐H1 IgM for type IIx (DHSB, 6‐H1‐supernatant), and Rabbit anti‐Laminin (1:150; Sigma, L9393) to identify cell membranes (Long et al., 2022; Walton et al., 2019). Sections were washed three times for 5 min with PBS and incubated for 1 h at room temperature with the following secondary antibody solution: Goat anti‐Mouse IgG2b Cross‐Adsorbed Secondary Antibody, Alexa Fluor 647 (1:250; Invitrogen, A‐21242), Goat anti‐Mouse IgG1 Cross‐Adsorbed Secondary Antibody, Alexa Fluor 488 (1:250; Invitrogen, A‐21121), Goat anti‐Mouse IgM (Heavy chain) Cross‐Adsorbed Secondary Antibody, Alexa Fluor 555 (1:250; Invitrogen, A‐21426), and AffiniPure Goat Anti‐Rabbit IgG (1:500; Jackson Immuno Research, 111‐065‐144). Following incubation, sections were washed three times for 5 min with PBS, then incubated for 1 h at room temperature with Steptavidin‐AMCA (1:200; Vector Laboratories, SA‐5008‐1), and mounted with 1:1 PBS/Glycerol and stored at 4°C.

To measure capillarization, sections were rehydrated by washing three times for 5 min with PBS. Sections were blocked with 2.5% normal horse serum for 1 h at room temperature, followed by a 90‐min incubation with an antibody solution of biotinylated Ulex Europaeus Agglutinin I (UEA I) (1:50; Vector, B‐1065‐2) and biotinylated Griffonia (Bandeiraea) Simplicifolia Lectin I (1:50; Vector, B‐1105‐2). Sections were then washed four times for 5 min with PBS and incubated with Streptavidin‐Alexa Fluor 488 (1:500; Thermo Scientific, S32354) for 1 h at RT. After the incubation, sections were washed three times for 5 min with PBS and incubated with DAPI (1:10,000; Thermo Scientific, D1306) for 10 min at room temperature (Kirkeby et al., 1993; Kosmac et al., 2020). Finally, sections were washed three times for 5 min with PBS and mounted with 1:1 PBS/Glycerol for storage at 4°C.

### 2.7 Muscle fiber image processing for analysis

All images were acquired using 20× magnification with a Zeiss AxioImager M1 upright microscope and analyzed using Zen Lite 3.4.9 software image processing computer software for Windows PC (Carl Zeiss AG, Oberkochen, Germany). Obtained images captured by the microscope were imported into the imaging processing software and allocated into appropriate channels based on muscle fiber type. Specifically, muscle fiber types and subsequent channels were allocated as Type I (Cy5), Type IIA (FITC), and Type IIX (TRITC). Borders surrounding all muscle fibers were indicated by laminin protein, which was realized by the DAPI channel. Original fluorescence of captured images from the microscope was shown as a grayscale image which was corrected manually by choosing the appropriate color for each channel to match the fluorescence observed through the microscope. Hence, each channel was reassigned as follows: Cy5 = pink, FITC = green, TRITC = red, and DAPI = blue. All uploaded images were enhanced for negative background and image contrast and brightness for optimal membrane immunofluorescence detection. Areas on the image subject to segmentation errors or non‐circular or longitudinal fibers were omitted using preexisting tools within the image processing software. Non‐circular and longitudinal fibers were omitted with the spline contour tool, and omitted areas were blacked out using the color correction tool to avoid detection from muscle fiber CSA and fiber typing quantification software. Enhanced images were exported into .PNG file format for use of automated high‐content analysis of muscle fiber CSA and fiber type distribution.

### 2.8 Muscle fiber CSA and fiber type analysis

Exported images from image processing software were uploaded into post‐processing muscle fiber CSA and fiber typing quantification computer software, MyoVision Basic (Version 2.0). MyoVision Basic provides high‐content quantification of muscle features, including fiber number, CSA and fiber type distribution without human supervision (Mula et al., 2013). For this study, MyoVision Basic was used to detect muscle fiber CSA (um^2^) and the fiber type (Type I, Type II, and Type IIx) for each muscle fiber. Muscle fiber CSA was computed using the fiber detection tool, which required a minimum area of 150 μm^2^ and a maximum area of 15,000 μm^2^ per image to appropriate human skeletal muscle tissue. Completion of fiber detection processing resulted in a newly contoured region of interest of the entire cross‐section for subsequent fiber type distribution analysis using the fiber typing tool. Muscle fiber CSA, fiber type, and shape data were exported to Microsoft Excel (Microsoft Corporation) for manual correction and omission of fibers falling outside parameters for circularity (≥0.60), solidity (≥0.85), and eccentricity (≥0.95) in accordance with established procedures (Wen et al., 2018). For fiber type analysis, fibers exhibiting IIa and IIx staining, IIax hybrid fibers, are included in IIa values.

### 2.9 Capillarization analyses

For the capillary analysis, images were exported in .TIFF format as an apropos input in Zeiss ZEN lite 3.0 imaging software (Zeiss). In an artifact‐free region, 50 contiguous muscle fibers were selected for analysis. Capillaries within the sample were manually counted using the event tool. Using the contour polygon tool, the cross‐sectional area of the 50‐muscle fiber sample was quantified. For each participant, for each time point, capillaries per fiber and capillary density (capillaries per total fiber area) were calculated for analysis.

### 2.10 Maximal strength

Squat, bench press, and deadlift maximal strength was assessed via one‐repetition maximums (1RMs) in accordance with National Strength and Conditioning Association testing protocols. For each movement, the participant was asked to estimate his or her 1RM after which the participant performed warm‐up sets of 8–10, 3–5, and 2–3 repetitions at approximately 50%, 75%, and 90% of the estimated 1RM, respectively. The initial 1RM attempt intensity was based on the participant's estimate and performance of the warm‐up sets. Following a successful 1RM attempt, squat and deadlift intensity was increased by 10%–20% while bench press intensity was increased by 5%–10%. Following an unsuccessful attempt, another 1RM was attempted at an intensity 5%–10% (squat and deadlift) or 2.5%–5% (bench press) lower. Attempts were performed until the 1RM was obtained. Two‐minute rest periods were provided between warm‐up sets and 1RM attempts.

### 2.11 Aerobic capacity

Participants completed an incremental Bruce treadmill protocol graded exercise test on a motorized treadmill (Woodway). The graded exercise test was performed fasted, following a minimum of 24 h of rest. The graded exercise test was performed to volitional failure with oxygen consumption measured via indirect calorimetry (Parvo TrueOne). Expired gases were sampled and analyzed breath by breath. Aerobic capacity was assessed as relative peak oxygen consumption (VO_2_peak). At least two of the following criteria had to be met to be considered a valid VO_2_peak, a plateau in oxygen consumption despite an increase in intensity, maximal HR within 10 beats per minute of the participant's age‐predicted maximal HR and a respiratory exchange ratio of ≥1.15.

### 2.12 Dual energy X‐ray absorptiometry

Lean body mass (LBM) was assessed via Lunar iDXA (GE Healthcare). Scans were performed following a minimum of 24 h rest, in a fasted state and in metal free, tight fitting clothing. Following manufacturer calibration procedures, a total body scan was performed in accordance with the manufacturer's guidelines for patient positioning and for scan acquisition (Shuhart et al., 2019). One total body scan was conducted for each participant. A repeat scan was performed only when the first scan included motion artifact or if a bodily region fell outside of the scan region. All scans were analyzed using enCORE Software, version 15 (GE Healthcare Lunar) by a Certified Bone Densitometry Technologist (CBDT). Regions of interest were adjusted as needed. Total body and leg region LBM measurements were used for statistical analyses.

### 2.13 Statistical analyses

Sex differences in the training‐induced changes in muscle fiber cross‐sectional area, muscle fiber type distribution, capillarization, mitochondrial biogenesis biomarker content (total PGC‐1α, citrate synthase and COX IV), lean body mass (total and leg region), maximal strength (squat, bench press and deadlift 1RMs), and aerobic capacity were assessed via two‐way (sex × time) ANOVAs. If significant interactions were observed, simple main effects of time at each level of sex were analyzed. If no significant interactions were observed, main effects of sex and time were reported and interpreted. Data are reported as mean ± standard deviation. Data analysis was conducted using IBM SPSS Statistics Version 28 (IBM Corporation). Alpha was set a priori at 0.05, two‐sided for all analyses.

## 3 RESULTS

In total, 433 men and women were screened for eligibility, and 247 met the inclusion criteria. Sixty‐six individuals were enrolled in the study; however, 14 participants voluntarily withdrew due to personal reasons and 13 were withdrawn by the study team due to non‐compliance with study procedures (*n* = 6), injuries sustained during study procedures (*n* = 3), injuries sustained outside of the study (*n* = 2), and because the research stopped during the COVID‐19 pandemic (*n* = 2). Of the three participants withdrawn due to study related injuries, one participant reported aggravating a previous low back injury during training and two participants experienced low back injuries during testing. Of the 39 individuals that completed the study, biopsies at both pre‐ and post‐training were obtained in 14 women (26 ± 5 yrs., 1.66 ± 0.06 m, 65.0 ± 11.7 kg) and 19 men (28 ± 4 years, 1.78 ± 0.09 m, 85.7 ± 14.6 kg). For the six individuals that completed the study but were not included in the muscle analyses, sufficient muscle tissue for analysis was not obtained at either of the time points or the participant abstained from biopsy procedures.

**FIGURE 2:**
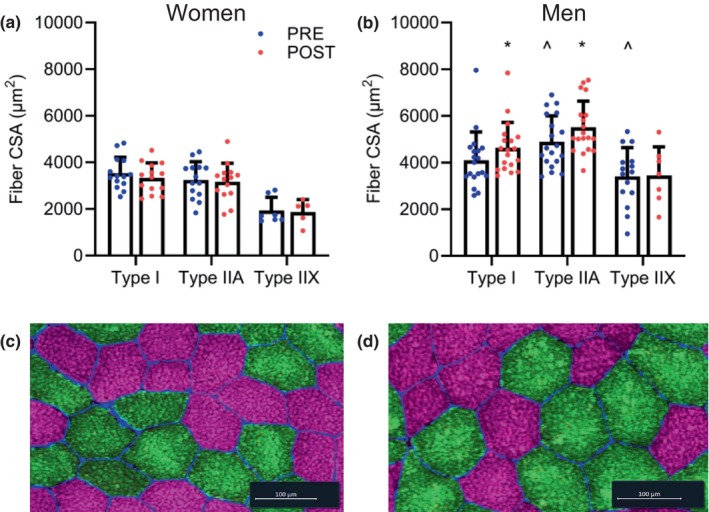
Skeletal muscle hypertrophy observed in men, but not women. Muscle fiber cross‐sectional area (CSA) values for women (a) and men (b) pre‐ and post‐training. Data are presented as individual data points and the group mean and standard deviation. *Significantly different from pre‐training, *p* < 0.05. ^Significantly different from women at pre‐training, *p* < 0.05. Representative images of a male participant demonstrating type I (pink) and type IIa (green) muscle fiber hypertrophy from pre‐ (c) to post‐training (d).

The training program elicited sex‐specific hypertrophy in type I and type IIa muscle fibers. Muscle fiber CSA data are presented in Figure 2. Significant sex × time interactions were observed for type I (*p* = 0.011) and type IIa (*p* = 0.022), but not type IIx (*p* = 0.899) muscle fibers. Post hoc analyses indicated no type I (*p* = 0.238) or type IIa (*p* = 0.696) muscle fiber hypertrophy in women. In contrast, men had a 12.9% increase in type I muscle fiber CSA (*p* = 0.016) and a 12.7% increase in type IIa muscle fiber CSA (*p* = 0.007). Prior to training, men had 50.7% greater type IIa (*p* < 0.001) and 102.2% greater type IIx (*p* = 0.016) muscle fiber CSA compared to women; however, type I muscle fiber CSA did not differ between sexes (*p* = 0.128).

Mean fiber type percentage values pre‐ and post‐training are reported in Table 1. Analyses of muscle fiber type distribution demonstrated no significant sex × time interactions (type I: *p* = 0.497; type IIa: *p* = 0.722; type IIx: *p* = 0.396). A main effect of time was observed for type IIx (*p* = 0.046) indicating a 1.9% decrease in type IIx fiber type percentage following training. Type IIx fibers were detected in 22 participants pre‐training, but only 12 participants post‐training. Main effects for time were not significant for type I (*p* = 0.316) or IIa (*p* = 0.803) fiber type percentage. Main effects of sex were not significant (type I: *p* = 0.063; type IIa: *p* = 0.058; type IIx: *p* = 0.448).

**TABLE 1:** Fiber type distribution of men and women pre‐ and post‐training.

	Type I (%)		Type IIa (%)		Type IIx (%)	
	PRE	POST	PRE	POST	PRE	POST
Women	47.2 ± 15.3	47.9 ± 13.6	50.9 ± 13.6	51.0 ± 15.7	2.0 ± 4.0	1.2 ± 2.6*
Men	34.6 ± 12.0	39.3 ± 10.2	62.2 ± 10.2	59.3 ± 9.8	3.2 ± 3.5	1.4 ± 4.4*

*Note*: Data are presented as mean ± SD.

* Significant main effect for time, *p* < 0.05.

Capillary count and density values are presented in Table 2. Capillary counts demonstrated no significant sex × time interaction (*p* = 0.124), nor main effect for time (*p* = 0.224). However, a significant main effect for sex (*p* = 0.004) indicated a greater number of capillaries per fiber in men than women. Capillary density demonstrated no significant sex × time interaction (*p* = 0.580), main effect for time (*p* = 0.121), or main effect of sex (*p* = 0.138).

**TABLE 2:** Capillary count and density in men and women pre‐ and post‐training.

	Capillary count (capillaries·fiber^−1^)		Capillary density (capillaries·mm^−2^)	
	PRE	POST	PRE	POST
Women	1.81 ± 0.43	1.63 ± 0.36*	386.0 ± 86.4	368.6 ± 81.2
Men	2.13 ± 0.42	2.15 ± 0.47	366.8 ± 75.7	330.5 ± 48.8

*Note*: Data are presented as mean ± SD.

* Significant main effect for sex, *p* < 0.05.

**FIGURE 3:**
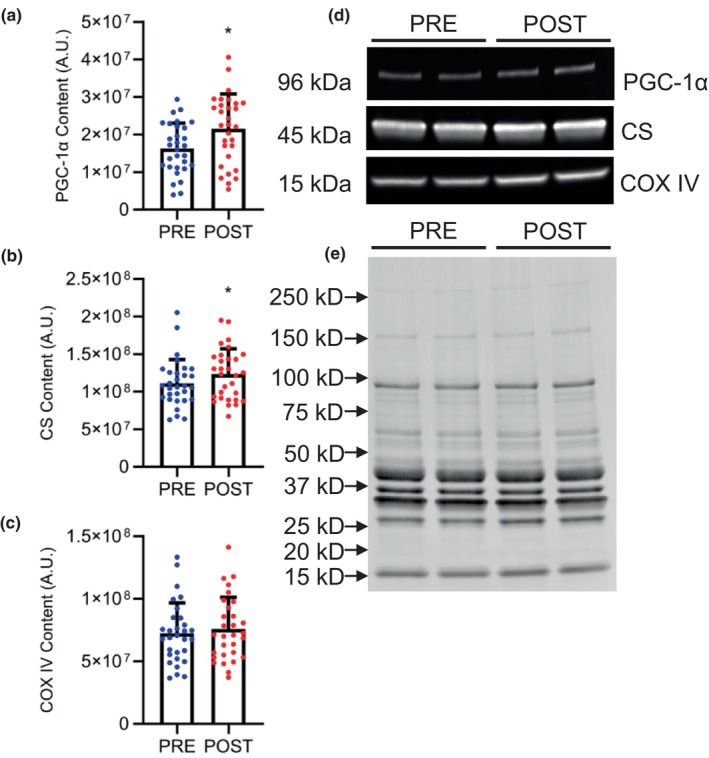
Markers of mitochondrial biogenesis including (a) peroxisome proliferator‐activated receptor gamma coactivator 1‐alpha (PGC‐1α), (b) citrate synthase (CS), and (c) cyotochrome c oxidase subunit IV (COX IV) at pre‐ and post‐training normalized to total protein. Data are combined for men and women. Data are presented as individual data points and the group mean and standard deviation. *Significantly different from pre‐training, *p* < 0.05. (d) Representative fluorescent western blot images for COX IV, CS, and PGC‐1α and (e) corresponding total protein blots for normalization.

Values for markers of mitochondrial biogenesis are presented in Figure 3. Total PGC‐1α demonstrated no significant sex × time interaction (*p* = 0.160) or main effect for sex (*p* = 0.893), but a significant main effect for time showed total PGC‐1α content increased 29.7% (*p* < 0.001) following training. Similarly, citrate synthase demonstrated no significant sex × time interaction (*p* = 0.768) or main effect for sex (*p* = 0.989), but a significant main effect for time showed citrate synthase content increased 11.0% (*p* < 0.014). COX IV demonstrated no significant sex × time interaction (*p* = 0.208), main effect for time (*p* = 0.295), or main effect of sex (*p* = 0.467).

**FIGURE 4:**
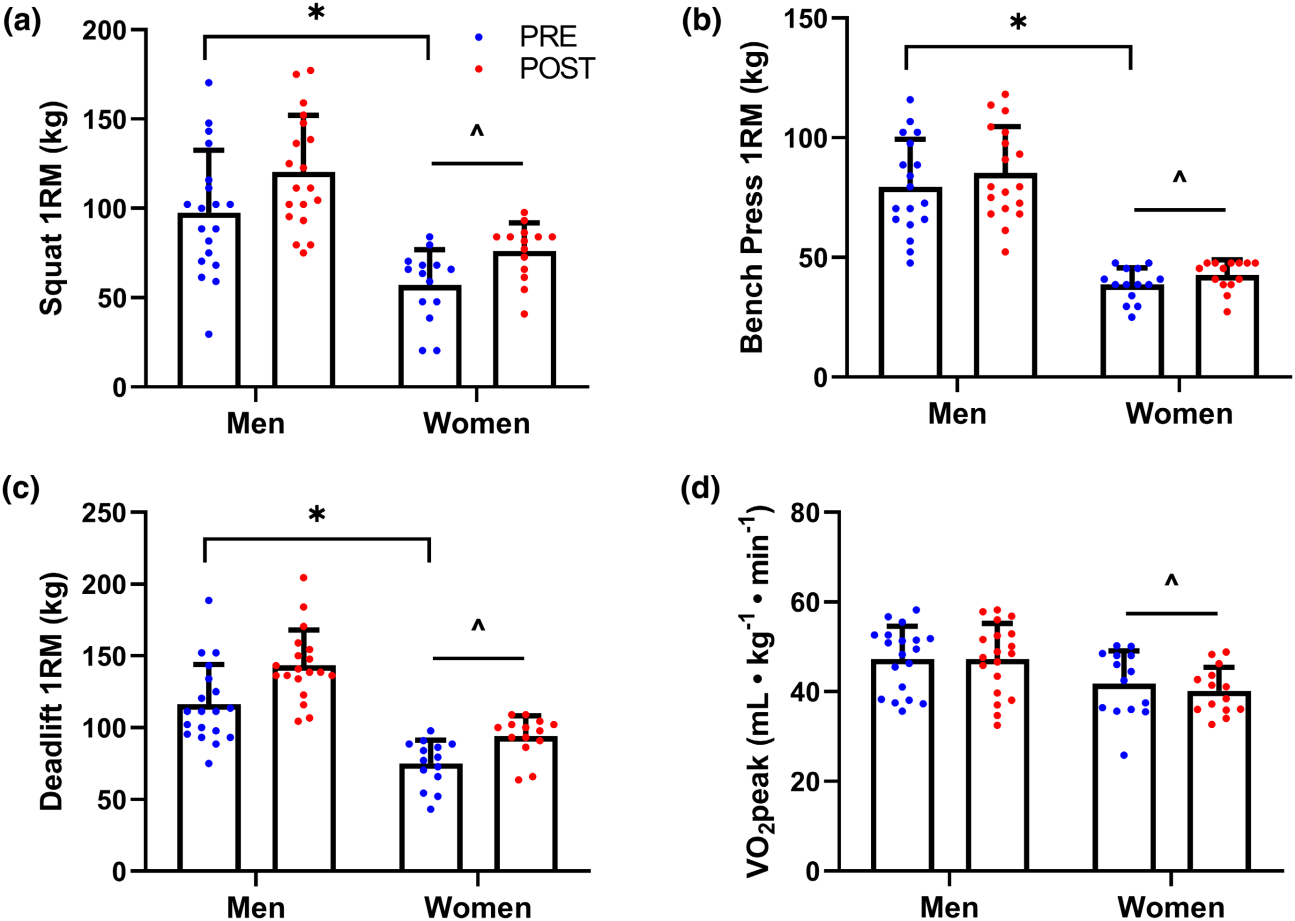
Pre‐ and post‐training physical performance in men and women including (a) squat one‐repetition maximum (1RM), (b) bench press 1RM, (c) deadlift 1RM, and (d) aerobic capacity. Data are presented as individual data points and the group mean and standard deviation. *Significant main effect for time, *p* < 0.05. ^Significant main effect for sex, *p* < 0.05.

No significant sex × time interactions were observed for squat (*p* = 0.404), bench press (*p* = 0.305), or deadlift (*p* = 0.058) maximal strength, or for VO_2_peak (*p* = 0.356). Main effects for time indicated that training increased squat, bench press, and deadlift maximal strength (*p* < 0.001), but not VO_2_peak (*p* = 0.363). Main effects for sex indicated that compared with women, men had greater maximal squat, bench press, and deadlift maximal strength and VO_2_peak (*p* < 0.001). Performance data are presented in Figure 4.

No significant sex × time interactions were observed for total body (*p* = 0.272) or leg (*p* = 0.164) LBM. Similar to performance outcomes, main effects for time indicated that training increased both total body (Pre: 52.4 ± 10.6 kg; Post: 54.2 ± 10.9 kg) and leg (Pre: 18.6 ± 4.3 kg; Post: 19.3 ± 4.4 kg) LBM (*p* < 0.001). Main effects for sex indicated that men had greater total body (Men: 60.6 ± 6.7 kg; Women: 43.4 ± 6.2 kg) and leg (Men: 21.7 ± 3.1 kg; Women: 15.3 ± 2.6 kg) LBM than women (*p* < 0.001).

## 4 DISCUSSION

The main findings of this study were that 12 weeks of concurrent resistance and interval training improved maximal strength, increased LBM, and reduced type IIx fiber type distribution in men and women. Despite similar increases in strength and LBM between sexes, only men demonstrated increased muscle fiber CSA post‐training, indicative of sex‐specific muscle fiber hypertrophy. Both sexes demonstrated small increases in markers of mitochondrial biogenesis and no change in muscle fiber capillarization, consistent with a lack of improvement in aerobic capacity.

In men, concurrent training elicited type I and IIa muscle fiber hypertrophy of 12.9% and 12.7%, respectively. No muscle fiber hypertrophy was observed in women, regardless of fiber type. Concurrent training‐induced muscle fiber hypertrophy in men is consistent with previous research (Kazior et al., 2016; Lundberg et al., 2013; Tsitkanou et al., 2017), but these study interventions have typically resulted in greater type II than type I muscle fiber hypertrophy. In the present study, similar type I and type IIa muscle fiber hypertrophy was observed. It is unlikely that similar fiber CSA increases observed in this study are the result of a concurrent training interference effect, as a recent meta‐analysis concluded that concurrent training may attenuate type I, but not type II muscle fiber hypertrophy (Lundberg et al., 2022). Instead, our fiber hypertrophy findings are more representative of lower‐intensity resistance training which can elicit greater type I than type II muscle fiber hypertrophy (Grgic & Schoenfeld, 2018; Mitchell et al., 2012). Differences in concurrent training program designs may explain the type I and type II fiber hypertrophy findings in men. Whereas other studies have predominantly employed high‐volume, high‐intensity resistance training with cycling or running based aerobic or interval training, the present training program included low‐volume resistance training with interval training based on jumps, runs/sprints, and weighted carries. These exercises were included due to their similarity to military relevant tasks and to reduce overall running volume, as high running volume is known to directly contribute to the prevalence of overuse‐associated musculoskeletal injuries in the military (Lovalekar et al., 2021; Nindl et al., 2013, 2016). It is possible that the relatively unique interval training program design represented more of a low‐intensity resistance training than an aerobic training stimulus, eliciting type I muscle fiber hypertrophy rather than mitochondrial biogenesis or angiogenesis. The muscle fiber and whole muscle hypertrophy may have been limited by the relatively low volume of the training program (Currier et al., 2023); however, optimizing hypertrophy must be weighed against the risks of overuse injury when programming the volume included in a training program intended for military populations.

In contrast with the current study, past research has reported female muscle fiber hypertrophy resulting from resistance and concurrent training programs (Bell et al., 2000; Binet et al., 2023; Roberts et al., 2020; Spiliopoulou et al., 2021; Staron et al., 1990). Although sex‐specific muscle fiber hypertrophy has been reported following resistance training interventions (Bamman et al., 2003; Martel et al., 2006; Moesgaard et al., 2022), a recent meta‐analysis concluded that there is no difference in the hypertrophic response to resistance training between men and women (Roberts et al., 2020). Therefore, while it is possible that the relatively unique concurrent training program with low‐volume resistance training was insufficient to stimulate muscle fiber hypertrophy in women (Terzis et al., 2016), the muscle fiber CSA findings may be due to the high variability in immunohistochemical analyses (Horwath et al., 2021). Furthermore, the similar increases in strength, total body, and leg LBM in men and women suggest that the concurrent training elicited similar muscle hypertrophy in both sexes. The poor agreement between increased fiber cross‐sectional area changes and DXA LBM changes are consistent with past research (Ruple et al., 2022).

Post‐training, increased total PGC‐1α and citrate synthase content may suggest that training increased skeletal muscle mitochondrial content in men and women. Alternatively, the greater post‐training total PGC‐1α content may be more indicative of increased mitochondrial respiration than increased mitochondrial content (Granata et al., 2016). Compared with previous studies demonstrating large increases in PGC‐1α (Konopka et al., 2014) and citrate synthase (Konopka et al., 2014; Skattebo et al., 2020) content, the ~30% increase in total PGC‐1α and 11% increase in citrate synthase in the current study are relatively small. The lack of change in COX IV despite the increase in citrate synthase may provide evidence of oxidative phosphorylation adaptations lagging behind adaptations to other metabolic processes such as the citric acid cycle, as has previously been reported (Granata et al., 2021). Aside from serving as a marker of mitochondrial biogenesis, PGC‐1α promotes muscle fiber type transition toward a more oxidative, less glycolytic fiber distribution (Lin et al., 2002). Thus, the increase in total PGC‐1α content and reduction in type IIx muscle fibers indicate a training‐induced transition toward a more oxidative fiber type distribution. Although a significant increase in type IIa fiber type was not detected, the decrease in IIx muscle fiber percentage is likely due to a transition of these fibers to a IIa or IIax hybrid muscle fiber type. Given that IIa and IIax are more oxidative and less glycolytic than type IIx, these fibers are more fatigue resistant (Herbison et al., 1982; Schiaffino & Reggiani, 2011).

Despite increased mitochondrial biogenesis marker content and a more fatigue resistant muscle fiber type, the training intervention had no effect on capillary count or capillary density in men or women, indicating limited angiogenic adaptation. The minor changes in mitochondrial biogenesis markers and lack of angiogenic adaptations are in accordance with the lack of improvement in aerobic capacity observed in the graded exercise test, indicating that the interval training was ineffective at improving aerobic performance. In contrast, sprint interval training has been shown to improve the number of capillary‐muscle fiber contacts and capillary density in men (Cocks et al., 2013, 2016). Notable differences in interval training design between the current study and previous high‐intensity interval training and sprint interval training studies likely contributed to the limited aerobic adaptations. Compared with previous studies, the interval training in the current study was performed at a lower intensity range than has previously demonstrated to elicit aerobic adaptations (MacInnis & Gibala, 2017). Additionally, past studies have employed running or cycling interval exercise, whereas the current study employed a variety of upper and lower body exercises with functional relevance to military tasks. Inclusion of both upper and lower body interval exercise limited lower body training volume, likely contributing to the lack of mitochondrial and angiogenic adaptations observed in the vastus lateralis.

For this study, participant dietary intake and non‐intervention physical activity were not controlled for, which potentially could have influenced the performance and skeletal muscle adaptations. Participants were instructed to maintain their normal dietary habits and limit non‐study physical activity throughout the training intervention. In female participants, menstrual cycle status and the use of hormonal contraception and were not controlled for. Menstrual cycle status could potentially have influenced performance outcomes; however, studies of the menstrual cycle's effect on performance have demonstrated inconsistent results (Colenso‐Semple et al., 2023), and a recent meta‐analysis concluded that the menstrual cycle does not influence strength performance (Blagrove et al., 2020). Our female participants included those who did (*n* = 7) and did not use hormonal contraception (*n* = 7). Given that hormonal contraception may impair hypertrophic adaptations to resistance training (Riechman & Lee, 2022), the lack of muscle fiber hypertrophy observed in women may partly be due to use of hormonal contraception by participants. While this study is underpowered to examine differences in muscle fiber hypertrophy between women who used hormonal birth control and those that did not, our data suggest that greater hypertrophy may have been observed in women who did not use hormonal birth control had our sample size been larger. Nonetheless, our study sample is representative of hormonal contraception use within the military (Enewold et al., 2010).

In conclusion, a high‐intensity, low‐volume concurrent training program elicited similar strength, lean body mass, and fiber type distribution adaptations in men and women. Only men demonstrated fiber hypertrophy in response to the training intervention; however, further research is necessary to determine whether this observation is due to the concurrent training program or due to the variability of muscle fiber CSA immunohistochemical analyses. The resistance training program successfully improved strength and increased lean body mass; however, the novel interval training was unsuccessful at improving aerobic capacity or eliciting aerobic adaptations in skeletal muscle.

## AUTHOR CONTRIBUTIONS

AJS, KTK, ML, SDF, CC, SLW, TJO, JPG, and BCN conceived and designed research. AJS, NRO, EDF, KTK, BJM, NMS, KJK, and CVE performed experiments. AJS, NRO, EDF, NMS, ML, and CKK analyzed data. AJS, ML, CKK, KJK, and BCN interpreted results of experiments. AJS prepared figures. AJS and NRO drafted manuscript. AJS, EDF, KTK, BJM, NMS, ML, CKK, KJK, SDF, CC, SLW, TJO, JPG, and BCN edited and revised manuscript. AJS and BCN approved final version of manuscript.

## FUNDING INFORMATION

This study was funded by the UK Ministry of Defense WGCC 5.5.6‐Task 0107.

## CONFLICT OF INTEREST STATEMENT

No conflicts of interest, financial or otherwise, are declared by the authors.

## ETHICS STATEMENT

All participants provided written informed consent following an explanation of risks and benefits associated with participation. The University of Pittsburgh Institutional Review Board (19030387) and United Kingdom Ministry of Defence Research Ethics Committee (903/MODREC/18) approved this study.

## Data Availability

Data will be made available upon reasonable request.
